# Navigating the Catholyte Landscape in All-Solid-State Batteries

**DOI:** 10.1021/acsenergylett.5c03429

**Published:** 2025-12-24

**Authors:** Julian F. Baumgärtner, Jaka Šivavec, Matthias Klimpel, Kostiantyn V. Kravchyk, Maksym V. Kovalenko

**Affiliations:** † Laboratory of Inorganic Chemistry, Department of Chemistry and Applied Biosciences, 27219 ETH Zürich, CH-8093 Zürich, Switzerland; ‡ Laboratory for Thin Films and Photovoltaics, Empa - Swiss Federal Laboratories for Materials Science & Technology, CH-8600 Dübendorf, Switzerland; § Institute of Energy Science and Technology (SIEST), Sungkyunkwan University (SKKU), 2066, Seobu-ro, Jangan-gu, Suwon, Gyeonggi-do 16419, Republic of Korea

## Abstract

All-solid-state batteries are widely regarded as the next frontier in electrochemical energy storage, offering the potential to surpass the energy density and safety limits of conventional lithium-ion batteries. Among the factors governing their performance, paramount are the choice and functionality of the solid-state electrolyte (SSE) as a catholyte within the composite positive electrode. This perspective critically examines the applicability and potential of the three most intensively studied inorganic SSE families, namely oxides, sulfides, and chlorides, as catholytes. We discuss their respective advantages, limitations, and compatibility with common cathode active materials, as well as the remaining knowledge gaps. We then assess whether any of the current SSE classes can be employed as a stand-alone SSE for all-solid-state batteries, or whether composite architectures combining multiple SSEs will ultimately be required.

## Graphical abstract

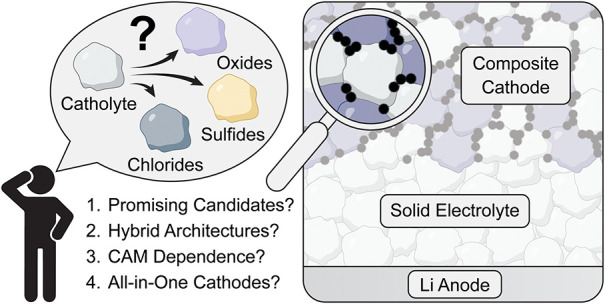

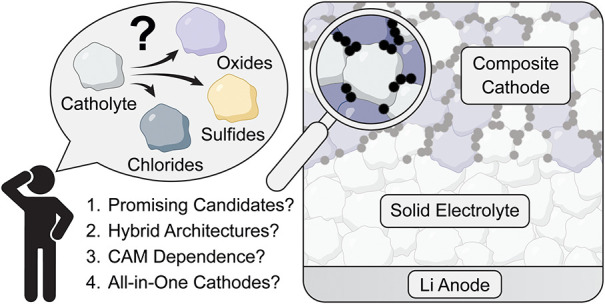

The commercialization of lithium-ion batteries (LIBs) by Sony in the early 1990s marked the beginning of a technological revolution that has driven advances in portable electronics, electric vehicles, and renewable energy storage.^1^ Yet, after decades of continuous optimization, LIBs are approaching their intrinsic limits in both energy density and safety. These constraints have motivated a global research shift toward solid-state batteries (SSBs) containing lithium metal anodes, widely regarded as the next frontier in electrochemical energy storage.^2^

Given the catastrophic failure of LIBs containing liquid electrolytes and a lithium metal anode,^3^ the reductive stability of solid-state electrolytes (SSEs) against lithium metal was initially considered the most critical challenge. The discovery of the reductively stable garnet-type SSE Li_7_La_3_Zr_2_O_12_ (LLZO), therefore, triggered a surge of research into inorganic SSEs.^4^ Around the same time, argyrodite-type lithium thiophosphates Li_6_PS_5_X (LPSX, X = Cl, Br, I) were reported to exhibit high ionic conductivities at room temperature (>2 mS cm^–1^),^5^ among which LPSCl showed particular promise owing to its ability to form a relatively stable solid-electrolyte interphase (SEI) with metallic lithium.^6^ More recently, lithium metal chlorides (LMCs) have emerged as a new class of SSEs with considerably higher oxidative stability than LPSCl, while offering comparable ionic conductivity and similar mechanical softness.^7^

Despite these advances, the optimal configuration of a SSB remains unclear. While the use of a lithium metal anode is widely accepted, and various strategies have been developed to stabilize its cycling behavior in contact with SSEs, the design of the positive electrode is far less resolved. The vast array of SSEs and cathode active materials (CAMs) gives rise to a multitude of possible CAM-SSE combinations, each with distinct interfacial and electrochemical challenges. Rational design of the positive electrode for practical SSBs thus remains a pertinent question, and is discussed in this Perspective. Specifically, we critically examine the fundamental applicability and potential of the most extensively studied inorganic SSE families, namely oxides, sulfides, and chlorides, as catholytes in SSBs.

The vast array of SSEs and cathode active materials (CAMs) gives rise to a multitude of possible CAM-SSE combinations, each with distinct interfacial and electrochemical challenges.

We aim to identify their respective advantages, limitations, and potential use cases, and to highlight the key knowledge gaps that continue to hinder progress. Furthermore, we explore whether any of the current SSE classes can realistically serve as a stand-alone component for fully solid-state architectures, or whether composite approaches that combine multiple SSEs to reconcile electrochemical and mechanical demands will ultimately be required, and what such designs might entail.

## Oxides

Out of all the oxidic SSEs discovered to date, LLZO is by far the most promising one. Yet even LLZO is only moderately conductive (0.3 mS cm^–1^) and has a high density (5.1 g cm^–3^). One may therefore rightfully question the feasibility of employing LLZO as a catholyte within a cathode composite. Early efforts were nonetheless justified by its exceptional (electro)­chemical stability against lithium metal. Theoretical predictions also suggested that, despite these intrinsic limitations, LLZO-based SSBs could still achieve competitive energy and power densities if higher SSE fractions (ca. 30 vol %) were incorporated within the cathode composite.^8^

However, subsequent studies emphasized severe practical challenges in fabricating LLZO-based cathodes, as co-sintering is required to establish intimate LLZO-CAM interfacial contact (Figure 1a). All state-of-the-art CAMs react chemically with LLZO at the high temperatures required for sintering (1100 to 1200 °C). For instance, LCO reacts with LLZO at temperatures as low as 700 °C, forming an ionically resistive La_2_CoO_4_ cathode-electrolyte interphase (CEI) that increases interfacial resistance and degrades cell performance.^9^ Co-sintering may also severely limit the usage of conductive carbon additives. For this reason, most studies investigate LCO as the CAM due to its intrinsically high electronic conductivity. Additionally, the mismatched thermal expansion between LLZO and most CAMs generates stresses that exceed the fracture strength of either component, causing cracks along the LLZO grain boundaries or the LLZO-CAM interface during cooling (Figure 1a).^10^

**Figure 1:**
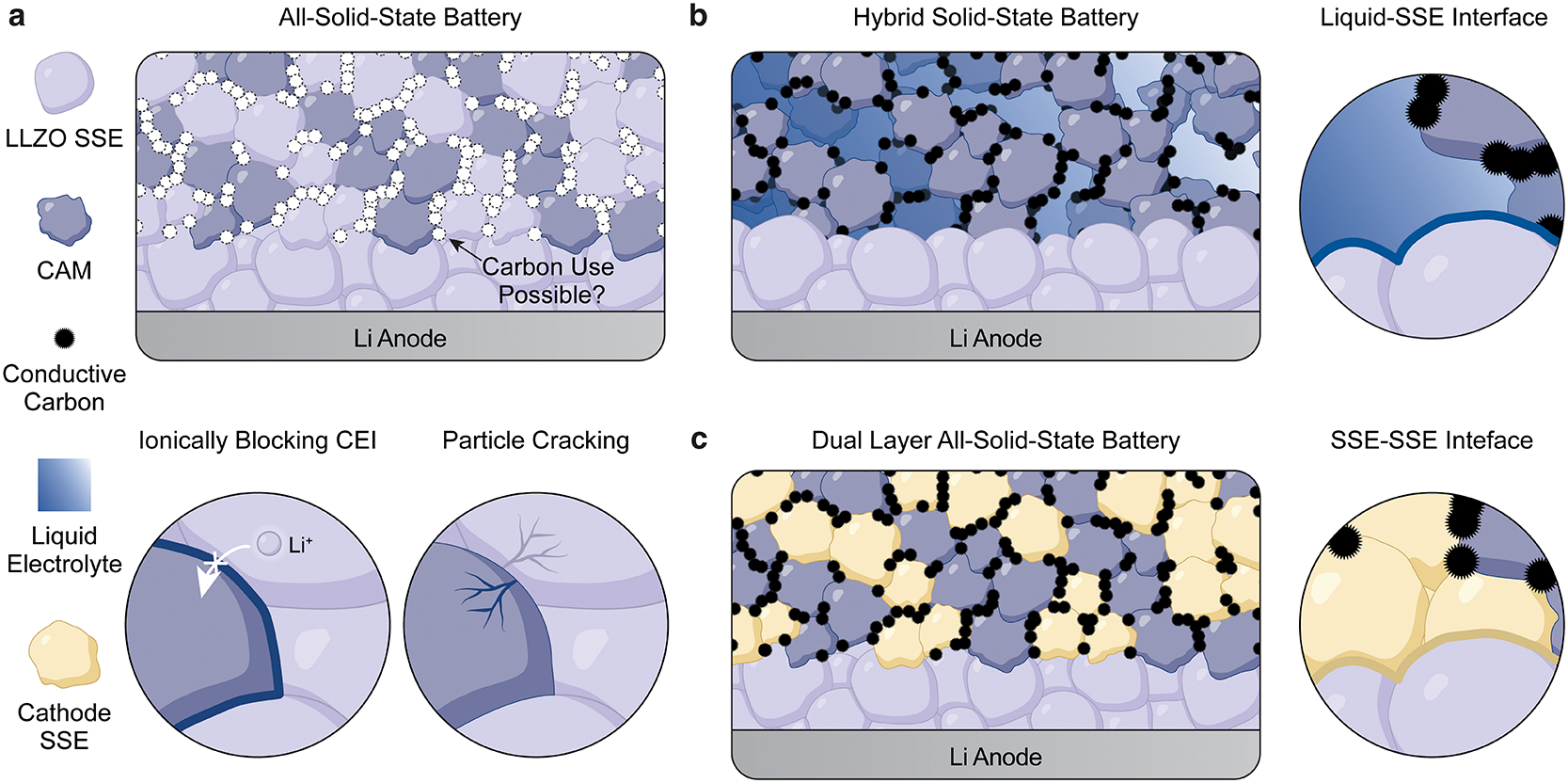
Possible architectures of LLZO-based SSBs. (a) Schematic of an LLZO-based ASSB. Cathode preparation by cosintering may preclude the usage of conductive carbon, resulting in the formation of an ionically blocking CEI and particle cracking. (b, c) Alternative SSB concepts based on a thin LLZO separator and a cathode composite containing a liquid (b) or solid (c) catholyte.

While LLZO-CAM interfacial reactions can be kinetically mitigated, for instance, by applying Al_2_O_3_ or LiNbO_3_ coatings on CAM particles^11^ or by shortening sintering times through ultrafast sintering,^12^ these strategies can not fully address the intrinsic challenges associated with LLZO sintering. Ultimately, reducing the sintering temperature of LLZO is essential. Sintering aids such as Li_2_CO_3_, Li_3_BO_3_, Li_2_SiO_3_, or Li_3_PO_3_ can form a liquid phase at lower temperatures, enabling densification below 800 °C. However, these additives reduce the overall energy density of ASSBs and, being relatively poor ionic conductors themselves, may disrupt ion transport within the cathode. Notably, the required reduction in sintering temperature to below 300 °C may be enabled by cold sintering under high pressure in a liquid environment, which had already been applied to LLZO.^13^

The significant challenges in fabricating LLZO-based cathodes are reflected in the limited electrochemical performance of most LLZO-based cathode composites. For example, ASSBs containing LCO as the CAM typically deliver capacities below 100 mAh g^–1^, and only a few reports demonstrate stable cycling beyond 20 cycles.^14^ The poor cyclability arises from volume changes in the CAM during (de)­lithiation, which induce cracking at the LLZO-CAM interface, leading to increased interfacial resistance and contact loss. Given that LLZO is a rigid SSE, applying high stack pressures provides only a slight improvement in compensating for CAM volume changes.

These practical fabrication challenges highlight that LLZO may not be suitable as a catholyte. Given its chemical stability against lithium metal, LLZO is more appropriately employed as a thin anode separator (<20 μm).^15^ Full-cell architectures could therefore integrate a thin LLZO separator paired with a lithium metal anode and a conventional cathode infiltrated with a liquid catholyte (Figure 1b,c), similar to the hybrid configuration adopted by QuantumScape, which is the only commercial manufacturer of ceramic-based SSBs.^16^ Such designs would also permit the use of less electronically conductive CAMs than LCO, such as NMC or LFP, and may further help mitigate cell polarization.^17^

Full-cell architectures could therefore integrate a thin LLZO separator paired with a lithium metal anode and a conventional cathode infiltrated with a liquid catholyte, similar to the hybrid configuration adopted by QuantumScape, which is the only commercial manufacturer of ceramic-based SSBs.

Importantly, most studies pursuing hybrid architectures have employed conventional carbonate-based electrolytes used in LIBs. However, carbonates chemically react with LLZO to form ionically resistive interphases.^18^ Since the catholyte is no longer in contact with the anode, alternative chemistries should be explored that ensure compatibility with both the CAM and LLZO while supporting high-voltage operation. For instance, promising results have been reported using ionic liquids,^19^ or deep eutectic solvents, such as LiTFSI in succinonitrile (Figure 1 b).^20^

Similarly, one could envision a full-cell configuration in which the LLZO separator is paired with a cathode incorporating another SSE, such as LPSCl or a LMC (*vide infra*).^21,22^ Owing to the mechanical softness of LPSCl or LMCs, effective SSE–SSE and SSE-CAM contacts could be achieved without the need for high-temperature sintering. However, the main challenge will be overcoming the formation of an ionically resistive interphases similar to the liquid cell (Figure 1 c).^21^ Alternatively, LLZO could be paired with a polymer to form a mechanically soft composite catholyte,^23^ or within a dual-layer approach.^24^

## Sulfides

Although LLZO represents the most mature oxide-based SSE, its limitations as a catholyte have shifted research focus toward other material classes, particularly sulfides, which offer distinct advantages in terms of ionic conductivity and interfacial contact. While novel sulfide SSEs are still being discovered, argyrodite-type LPSX, and in particular LPSCl is currently the most promising one, because of its ability to form a relatively stable SEI that enables reversible lithium plating and stripping despite the narrow thermodynamic stability window of only 1.5 to 2.5 V vs Li^+^/Li. In contrast, oxidative decomposition of LPSCl at the LPSCl-CAM interface, especially at high states of charge, leads to a growing CEI and steadily increasing interfacial impedance.^25^ Consequently, much of the research on LPSCl-based SSBs has focused on understanding and mitigating reactions at the LPSCl-CAM interface.

Most of these studies have explored state-of-the-art CAMs, such as LCO or NMC. Protective CAM coatings originally developed for liquid electrolyte systems, including LiNbO_3_ and LiAlO_2_, have proven effective in suppressing interfacial degradation between LPSCl and oxide-based cathodes, particularly during high-voltage operation.^10^ With such coatings, NMC-based LPSCl ASSBs demonstrated high cycling stability at commercially relevant areal capacities (6.8 mAh cm^–2^) and cycling rates (2C), showcasing that LPSCl can indeed be used as a singular SSE in commercial cells (Figure 2a).^26^ Despite these advances, the dependence on extensive CEI engineering underscores the need for SSEs with inherently greater oxidative stability (*vide infra*), as well as for a deeper understanding of how a beneficial CEI could be formed *in situ*.

**Figure 2:**
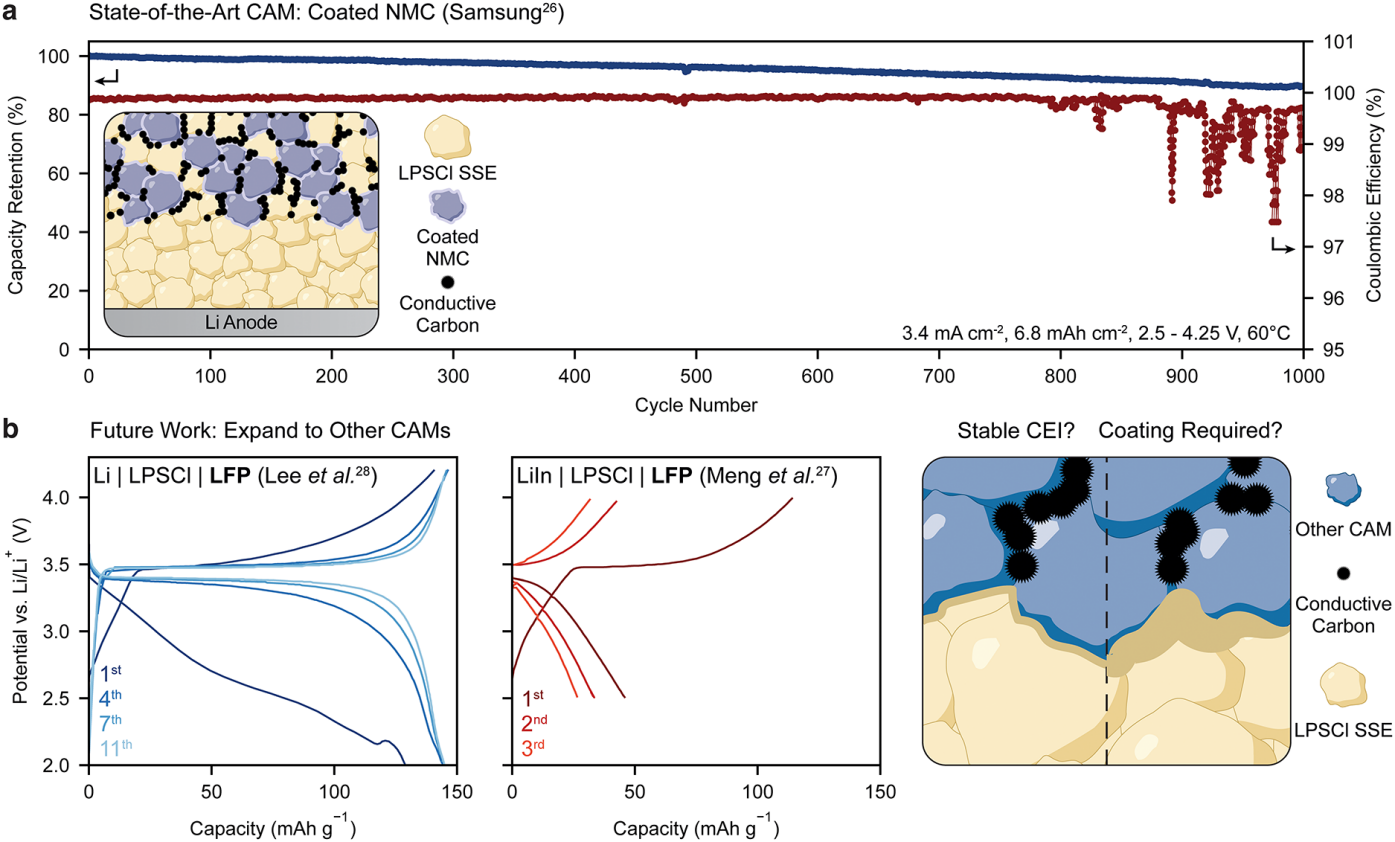
Perspectives on LPSCl-based SSBs. (a) State-of-the-art electrochemical performance of LPSCl-based ASSBs with coated NMC CAM (Adapted with permission.^26^ Copyright 2020, Springer Nature). (b) Differing reports from Lee et al. (Adapted with permission.^28^ Copyright © 2022 American Chemical Society) and Meng et al. (Adapted with permission.^27^ Copyright © 2023 American Chemical Society) the voltage profiles of LPSCl-based cells with LFP, highlighting the need for understanding the compatibility with other CAMs.

Importantly, the performance of LPSCl-based ASSBs incorporating alternative CAMs also remains largely unexplored (Figure 2b). Considering the projected surge in battery demand, it is essential to identify CAMs that balance not only energy density but also cost and sustainability in practical cell architectures. In this regard, Fe-based CAMs are particularly attractive due to the high abundance of iron on Earth. Yet, despite its widespread use in low-cost LIBs, LFP has rarely been studied in combination with LPSCl. Meng et al.^27^ reported poor electrochemical performance of LFP in LPSCl-based ASSB, with low specific capacities and rapid capacity fade within ten cycles, attributed to oxidative decomposition of LPSCl accompanied by severe microstructural degradation of the cathode composite (Figure 2b). This led the authors to conclude that LPSCl and LFP are intrinsically incompatible. Somewhat surprisingly, other studies have demonstrated LFP-LPSCl composites delivering initial capacities of 141 mAh g^–1^ and excellent capacity retention over 1000 cycles, but only if LPSCl with small particle sizes were used.^28^ Despite promising performance and the clear contradictions in the literature regarding LFP-LPSCl compatibility, no detailed follow-up studies were performed on this system.

Despite promising performance and the clear contradictions in the literature regarding LFP-LPSCl compatibility, no detailed follow-up studies were performed on this system.

Other compelling yet less established low-cost CAMs remain similarly underexplored (Figure 2b). Iron fluorides, for instance, exhibit high specific capacities and stable cycling performance in liquid electrolytes,^29^ and their pairing with SSEs is especially appealing given their Li-deficient nature, which renders lithium metal an ideal anode counterpart. The stack pressure typically applied in ASSBs could further mitigate the contact loss that results from large volume changes during (de)­lithiation of fluoride-containing CAMs. Regardless, only a handful of reports examined the performance of FeF_*x*_-LPSCl composites. Han et al.^30^ found that conversion-type FeF_2_ reacts chemically with LPSCl, resulting in SSE decomposition accompanied by FeS species formation, consistent with recent findings on a related pyrochlore-type iron hydroxy fluoride, where oxidative decomposition of LPSCl was identified as the primary cause of cathode degradation.^31^ While these findings suggest that FeF_*x*_-LPSCl composites are inherently incompatible, other studies have demonstrated the cycling stability of FeF_*x*_-LPSCl composites for up to 400 cycles when heat-treated FeF_3_ is employed.^32^ Although it remains unclear whether the observed capacity originates solely from Fe redox, or also involves SSE redox contributions, these results highlight that FeF_*x*_-LPSCl systems warrant more comprehensive investigation.

Taken together, these inconsistencies reflect the field’s strong focus on high-energy density CAMs, while other cathode chemistries remain far less studied. Yet, broadening this scope and developing a better mechanistic understanding of the interfacial behavior with other CAMs could reveal solutions comparable to protective coatings for NMC, and ultimately expand the range of viable CAM-LPSCl combinations for diverse energy storage applications.

## Chlorides

As discussed above, the lack of chemical compatibility and the need for extensive CEI engineering in LPSCl-based ASSBs have motivated the continued exploration of SSEs with inherently greater (electro)­chemical stability toward a range of CAMs. In this respect, LMCs have recently emerged as a compelling new class of SSEs,^7^ since the high electronegativity of Cl gives rise to an oxidation potential of ca. 4 V vs Li^+^/Li.

Unlike for oxides and sulfides, where LLZO and LPSCl are widely accepted as the most promising candidates, no clear candidate has emerged for chlorides yet, given the relatively nascent stage of research (Figure 3a,b). Researchers are still exploring diverse cationic (e.g., In, Y, Nb, Ta, Ho, Sc, Y and Zr) and anionic substitutions (e.g., Br, O), alongside various synthesis routes. These factors strongly affect ionic conductivity, (electro)­chemical stability, cost and density of LMC. Y- and Zr-based LMC are favored for cost and density,^33^ whereas those with In and Sc show the highest ionic conductivities, reaching up to 2 mS cm^–1^. Strategies such as mixed bromo-chlorides,^34^ cation (dis)­order,^35^ metal ratio,^36^ or non-close-packed chloride sublattices^37^ are promising avenues to improve the ionic conductivity. An auspicious approach to further enhance the ionic conductivity is through partial anion substitution with O to form LiMOCl_4_.^38^ This incorporation of O and high-valent cations results in non-closed-packed structures with vastly improved ionic conductivities exceeding 10 mS cm^–1^.

**Figure 3:**
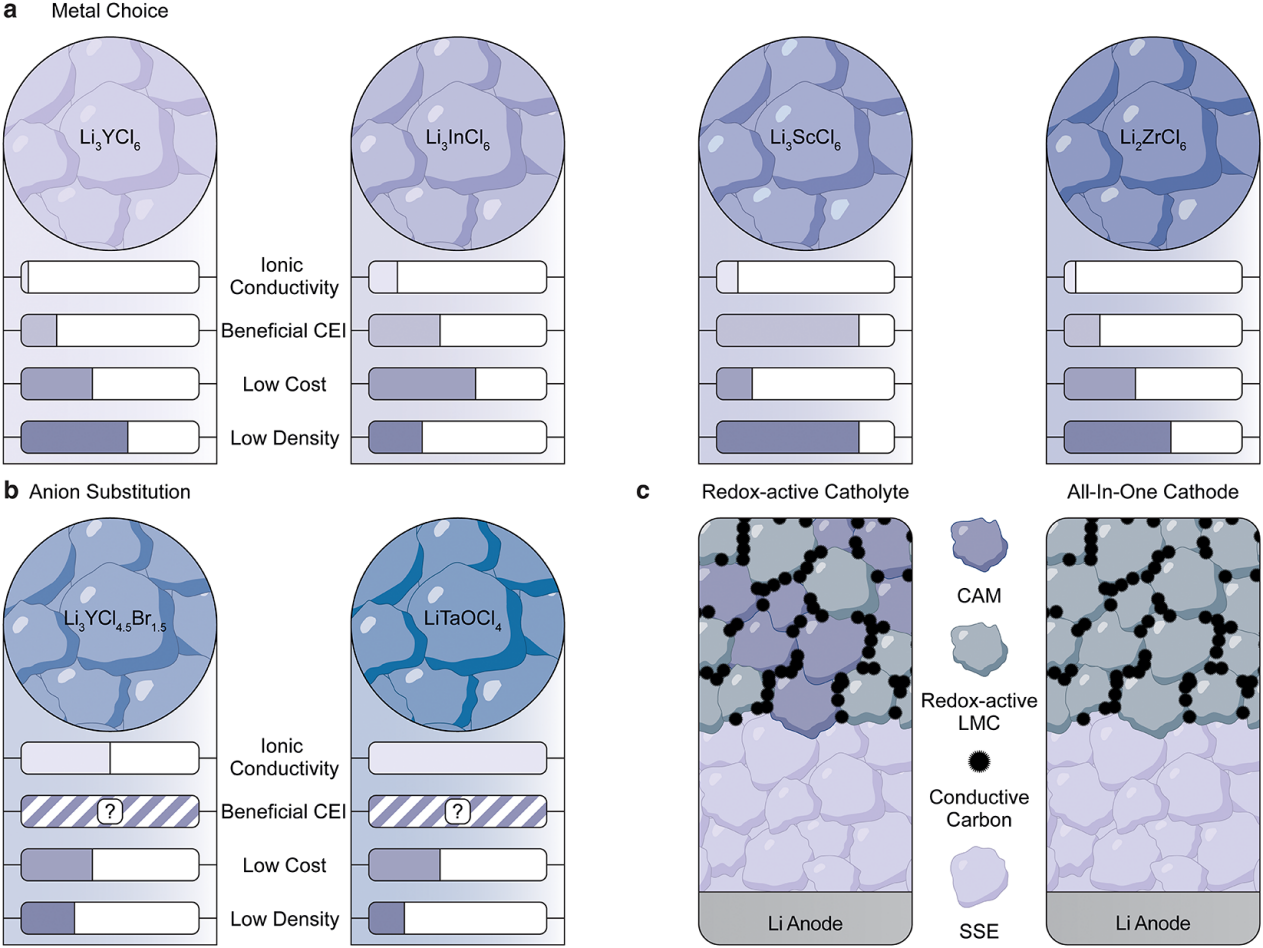
Strategies for Chloride-based SSBs. (a, b) Comparison of LMCs with different metals (a) and partial anion substitution (b) for their use as catholytes. (c) Schematic of ASSBs containing redox-active LMCs with mixed ionic-electronic conductivity as catholytes or as all-in-one cathodes.

While the intrinsically higher oxidative stability of chlorides compared to sulfides goes a long way to overcoming the compatibility issues of inorganic SSEs with CAMs, a gap of 0.3–0.6 V remains between the desired voltage cutoff of the SSB and the intrinsic stability window of chlorides. The formation of a favorable CEI is therefore required to extend the practical voltage window of chloride-based ASSBs. Just like sulfides and oxides, chlorides react chemically with oxide-based CAMs to form a CEI composed of oxides and oxychlorides, the properties of which crucially depend on the choice of metal in the LMC. In and Sc-based LMCs form a kinetically stable CEI, allowing cell operation up to 4.6 V, while those containing Y and Zr form a more reactive CEI, and consequently require a lower voltage cutoff.^39^ To what extent protective CAM coatings could be used to extend the oxidative stability in a similar way is also not well researched yet.

An important caveat that must be kept in mind is that the carbon additives within the cathode composite do not form a favorable interface with the SSE and cause SSE decomposition at lower voltages.^40,41^ This may force a compromise for cathode fabrication between electronic conductivity and interfacial stability, which may, however, be minimized by the usage of low surface area carbon, or possibly oxygen-functionalized carbon additives that could form interfaces comparable to CEIs.

One of the more creative ways in which chlorides could transform SSB cathodes is by incorporating redox-active transition metals, e.g. Fe,^42–44^ V,^45^ or Ti,^46^ into the otherwise redox-inactive, electronically insulating chloride structure. This may result in a redox-active catholyte that retains the favorable ionic conductivities and ductilities (Figure 3c). Such systems could function either as all-in-one electrodes or in composites with oxide-based CAMs to significantly enhance the energy and power density of ASSBs by lowering the Li-ion diffusion path tortuosity.^43^ Yet, at the same time, the absence of any favorable CEIs in an all-in-one electrode may limit their practical voltage window to about 4 V vs Li^+^/Li.

One of the more creative ways in which chlorides could transform SSB cathodes is that by incorporating redox-active transition metals, e.g. Fe,^42–44^ V,^45^ or Ti^46^, into the structure, the otherwise redox-inactive, electronically insulating chlorides may be converted into a redox-active catholyte that retains the favorable ionic conductivities and ductilities.

Contrary to LLZO or LPSCl, it is still unclear how lithium metal stability can be achieved in LMCs due to the propensity of the transition metal (e.g., In, Y, Nb, Ta, Ho) to be reduced by metallic lithium,^47^ and in some cases even by LiIn. This problem is exacerbated by the formation of metallic, electronically conductive decomposition products, leading to a continuous growth of the SEI, and subsequent decomposition of the electrolyte. However, there have been compelling reports on the formation of a favorable SEI with metallic lithium for La- and Ta-containing LMCs,^48^ as well as for mixed bromo- or iodo-chlorides.^49,50^ If the same LMC would also form a beneficial CEI, this may enable a SSB comprised of a single SSE, much like with sulfides. Otherwise, LMC would be best suited as a pure catholyte.

## Summary and Outlook

Notwithstanding the considerable progress achieved in the exploration of various SSEs and their use cases in SSBs, the path to the commercialization of SSBs remains arduous. Among the currently studied SSEs, LPSCl appears to be the most compelling and mature one, suitable for use both as a catholyte and as a separator in contact with the lithium metal anode (Figure 4). To date, its performance has been evaluated primarily with NMC. Future research should therefore expand the scope of LPSCl to other viable CAMs, employing optimized coating strategies to mitigate oxidative degradation upon contact with these CAMs.

**Figure 4:**
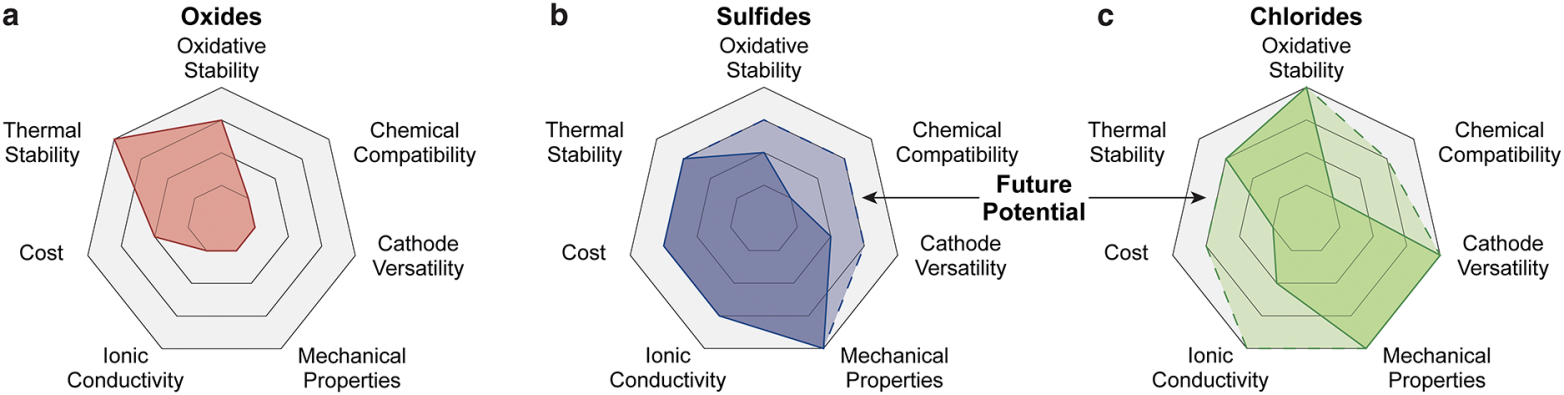
Comparison and potential of different SSEs as catholytes. Radar plots with the relevant properties for oxides (a), sulfides (b) and chlorides (c). Dashed areas indicate potential for further improvements through more comprehensive studies.

In contrast, the use of LLZO as a catholyte appears less compelling. Given the demonstrated electrochemical performance of LPSCl-based cathodes and the severe processing challenges associated with LLZO, this SSE is unlikely to become a competitive catholyte, regardless of the CAM. Nevertheless, LLZO may still find a niche as a robust anode-side separator within hybrid SSBs that combine a Li anode with a liquid- or solid-based cathode. Although such hybrid architectures introduce additional interfaces, their commercial potential has already been demonstrated, most notably by QuantumScape.

Research on LMCs is still at an earlier stage compared to sulfides, but their intrinsic properties already indicate strong potential as a catholytes for a wide range of CAMs, particularly because they may not require cathode coatings. A substantial leap in both energy and power density could be achieved through the development of redox-active LMCs, for instance, those incorporating transition metals such as Fe, which could simultaneously function as both catholyte and CAM. Currently, the main limitation of LMCs is their instability in contact with the lithium metal anode, restricting their use to the cathode side in hybrid architectures. Overcoming this limitation, either by interface engineering or through hybrid cell design, will be a critical step toward realizing their full potential.
